# Cortical thickness of the tibial diaphysis reveals age- and sex-related characteristics between non-obese healthy young and elderly subjects depending on the tibial regions

**DOI:** 10.1186/s40634-020-00297-9

**Published:** 2020-10-06

**Authors:** Keisuke Maeda, Tomoharu Mochizuki, Koichi Kobayashi, Osamu Tanifuji, Keiichiro Someya, Sho Hokari, Ryota Katsumi, Yusuke Morise, Hiroshi Koga, Makoto Sakamoto, Yoshio Koga, Hiroyuki Kawashima

**Affiliations:** 1 Division of Orthopedic Surgery, Department of Regenerative and Transplant Medicine, Niigata University Graduate School of Medical and Dental Science, 1-757 Asahimachi-dori Chuo–ku, Niigata City, Niigata, 951-8510 Japan; 2 School of Health Sciences, Faculty of Medicine, Niigata University, Niigata, Japan; 3 Graduate School of Science and Technology, Niigata University, Niigata, Japan; 4 Department of Orthopedic Surgery, Nioji Onsen Hospital, Niigata, Japan

**Keywords:** Age, Sex, Cortical bone, Tibia

## Abstract

**Purpose:**

This study aimed to evaluate the age- and sex-related characteristics in cortical thickness of the tibial diaphysis between non-obese healthy young and elderly subjects as reference data.

**Methods:**

The study investigated 31 young subjects (12 men and 19 women; mean age, 25 ± 8 years) and 54 elderly subjects (29 men and 25 women; mean age, 70 ± 6 years). Three-dimensional estimated cortical thickness of the tibial diaphysis was automatically calculated for 5000–9000 measurement points using the high-resolution cortical thickness measurement from clinical computed tomography data. In 12 assessment regions created by combining three heights (proximal, central, and distal diaphysis) and four areas of the axial plane at 90° (medial, anterior, lateral, and posterior areas) in the tibial coordinate system, the standardized thickness was assessed using the tibial length.

**Results:**

As structural characteristics, there were no differences in the medial and lateral thicknesses, while the anterior thickness was greater than the posterior thickness in all groups. The sex-related difference was not shown. As an age-related difference, elderly subjects showed greater or lesser cortical thickness than the young subjects, depending on the regions of the tibia.

**Conclusions:**

Cortical thickness was different depending on sex, age, and regions in the tibia. The results of this study are of clinical relevance as reference points to clarify the causes of various pathological conditions for diseases.

**Level of evidence:**

Level 3.

## Introduction

Three-dimensional cortical bone thickness varies depending on the regions of the bone [1] and is the optimal parameter to evaluate structural adaptation by biological factors and mechanical use [2]. A bone’s resistance is dependent on the sex- and age-related characteristics of cortical thickness [3, 4], which is useful in determining the causes of various pathological conditions, such as knee osteoarthritis (OA) [5–11].

Our group has been conducting an epidemiological study with regard to knee OA in Matsudai district of Tokamachi City in Niigata Prefecture in seven-year intervals since 1979 (Matsudai Knee Osteoarthritis Survey) [12–18]. The epidemiological study has the advantage of clarifying the sequence of changes and causes of initiation and progression. This cohort demonstrated that tibial cortical thickness is one of the key factors in clarifying the etiology of knee OA [5].

**Fig. 1:**
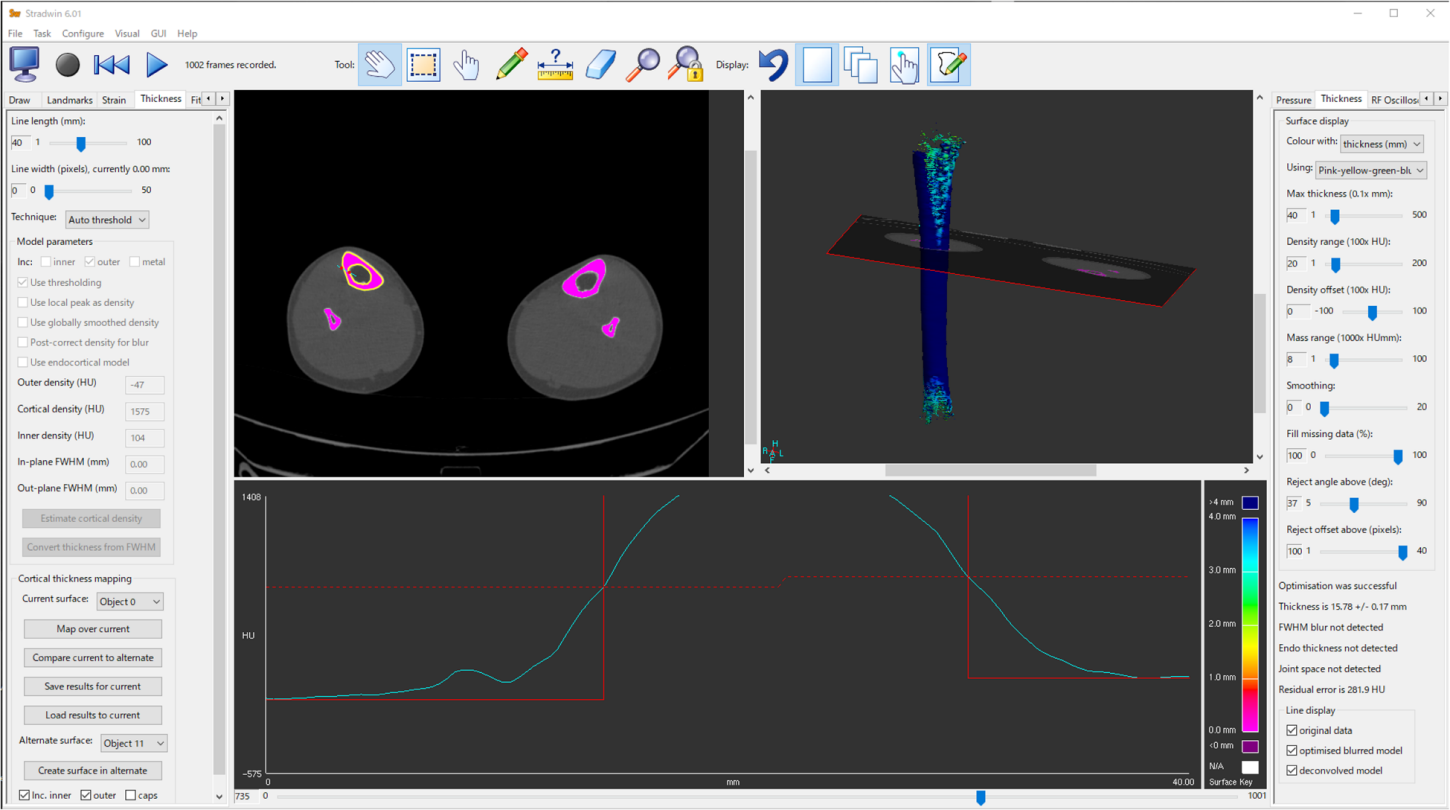
Automatic calculation using the high-resolution cortical thickness measurement from clinical CT data. The technique relies on a mathematical model of the anatomy and imaging system, which is fitted to the data at a large number of sites around the femur. Given prior segmentation of the tibial diaphysis, CT values are examined along short lines that straddle and are perpendicular to the cortex

**Fig. 2:**
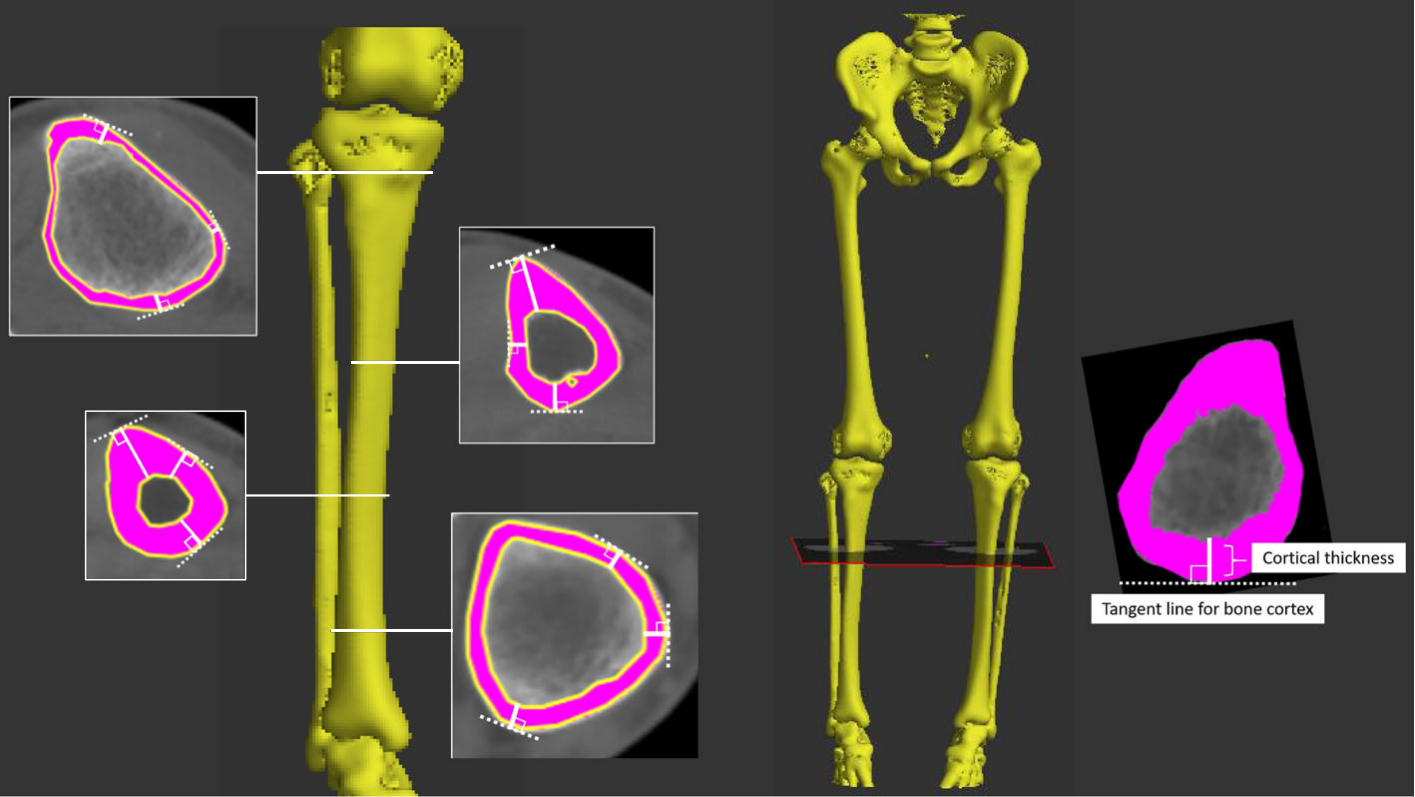
Schematic illustration of the three-dimensional bone models and tibial cortical thickness mapped back onto the surface as a color. Cortical thickness of the tibia can be mapped across the entire surface of the tibia using a cortical mapping technique

Measuring cortical thickness and volumetric density is challenging by clinical low-resolution computed tomography (CT). Simple thickness estimates based on several measures [19–22] have not provided optimal measurements. To date, Treece et al. [23, 24] reported highly accurate measurements by which cortical thickness can be accurately estimated from the clinical low-resolution CT data, using the mathematical model of the anatomy and imaging system (Figs. 1 and 2) [23, 24].

Data regarding cortical thickness to evaluate bony deformity and to elucidate the etiology of pathological conditions in the lower extremity, such as knee OA, is important. A previous study reported the age- and sex-related characteristics of cortical thickness in the femoral diaphysis for healthy subjects as reference data [1]; however, control data of the tibial diaphysis remains unknown.

This study aimed to identify the age- and sex-related characteristics of three-dimensional cortical thickness of the tibial diaphysis in healthy non-obese young and elderly individuals, using high-resolution cortical thickness measurement from clinical CT data. The hypothesis was that the cortical thickness of the tibial diaphysis would show age- and sex-related characteristics depending on the tibial regions.

## Materials and methods

### Subjects

The inclusion criteria were healthy elderly (age > 50 years) and healthy young (age < 50 years) subjects who were not obese [body mass index (BMI) < 30]. The exclusion criteria were not subjects with obesity (BMI > 30), history of trauma, knee OA, or other diseases that influence cortical thickness, such as osteometabolic diseases, except for primary osteoporosis.

**Fig. 3:**
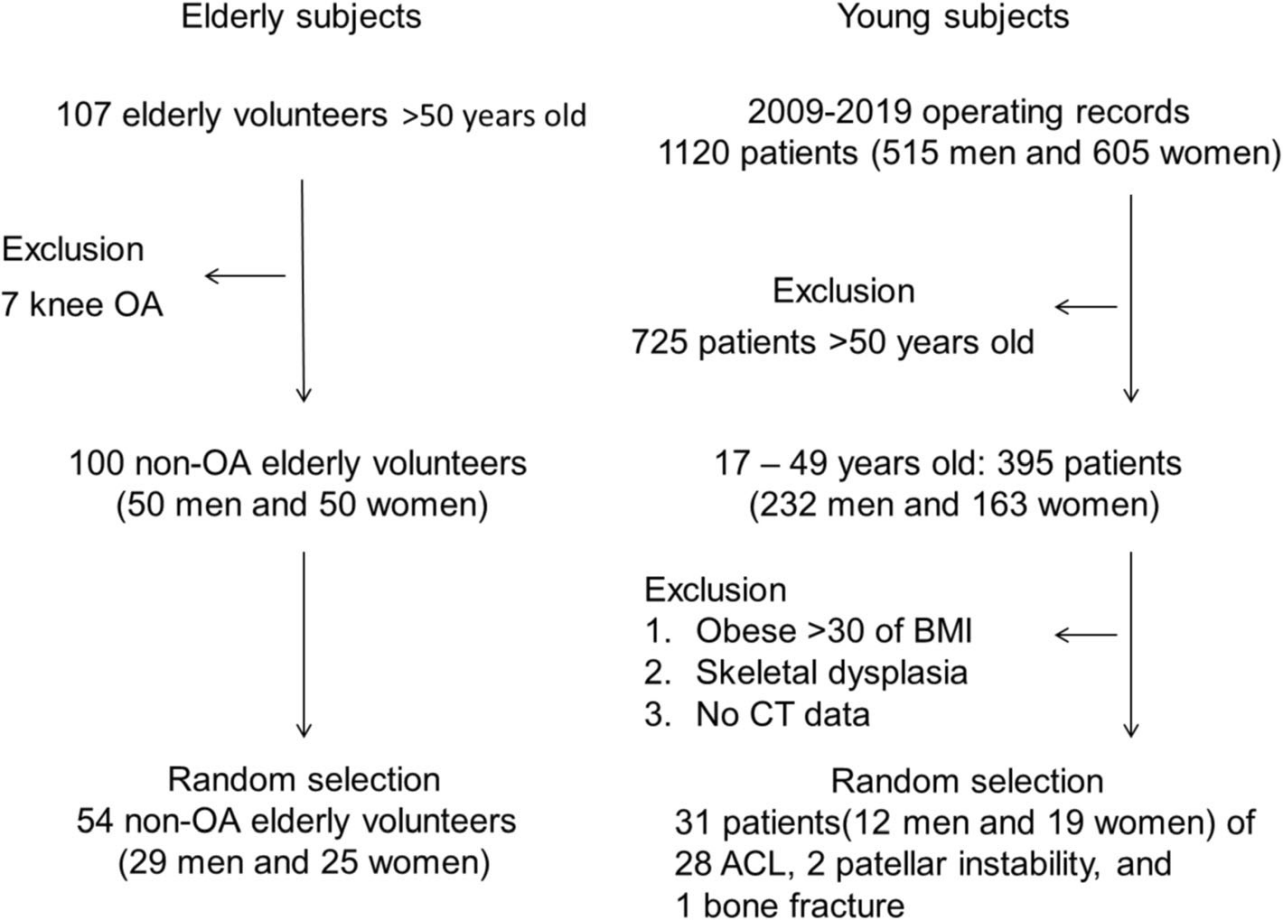
Inclusion and exclusion criteria of the subjects. OA: osteoarthritis, BMI: body mass index, ACL: anterior cruciate ligament

First, a total of 107 elderly Japanese volunteers who had no knee complaints or histories of joint disease or major injury in the lower extremity were publicly recruited. The volunteers did not have any competing interests and were not paid fees. Physicians assessed their general and lower extremity conditions using physical tests and radiographs and excluded seven subjects with radiographic evidence of knee OA. Out of 100 healthy elderly patients (50 men and 50 women) with grades 0–1 on the Kellgren–Lawrence (K–L) classification and absence of radiographic knee OA, 54 elderly (aged > 50 years) Japanese volunteers (29 men and 25 women) were randomly selected in this study (Fig. 3). Two orthopedic surgeons (graders) who were not provided with any clinical information of the patients performed the K–L classification. When the same subject was assigned different grades, the graders discussed and determined a common K–L grade. The average age ± standard deviation (SD) (range) of the elderly men and women was 71 ± 6 years (61 to 83 years) and 68 ± 5 years (60 to 83 years), respectively. The average BMI ± SD (range) of the elderly men and women was 23.0 ± 2.0 kg/m^2^ (17.6 to 27.0 kg/m^2^) and 20.0 ± 1.6 kg/m^2^ (17.1 to 23.0 kg/m^2^), respectively.

In young participants (age < 50 years), 395 out of 1120 patients were initially selected from the medical operating records of 2009–2019. Obese participants (BMI > 30), those with diseases that influence cortical thickness, such as skeletal dysplasia, infection, and osteometabolic disease, and those with no CT or BMI data were excluded. Finally, the lower extremities of 31 healthy young Japanese participants (12 men and 19 women) were randomly selected from 395 patients aged < 50 years using the abovementioned exclusion criteria. Data from the contralateral side of the patients with 28 anterior cruciate ligament injuries, two patellar instabilities, and one traumatic bone fracture are included in Fig. 3. The average age ± SD (range) was 26 ± 6 years (20 to 39 years) and 25 ± 9 years (17 to 41 years) in the young men and women, respectively. The average BMI ± SD (range) was 22.1 ± 1.4 kg/m^2^ (20.0–24.1 kg/m^2^) and 22.2 ± 2.6 kg/m^2^ (17.7–27.7 kg/m^2^) in young men and women, respectively.

### CT scanning condition

CT scans with a 1-mm interval in the lower extremities from the femoral head to the ankle joint were performed in two institutions, for the young subjects using Somatom Sensation 16 (Siemens Inc., Munich, Germany) in our university and for the elderly subjects using Canon Aquilion 64 CT scanner (Canon Medical Systems, Tochigi, Japan) in the other institution. The scans were obtained at a tube voltage of 120 kVp and current of 50–400 mA. The field of view, matrix, and pixel parameters were 350–400 mm, 512 × 512, and 0.68–0.78 mm/pixel, respectively. The voxel size was 0.68 × 0.68 × 1.00 mm to 0.78 × 0.78 × 1.00 mm. For CT radiation, the mean dose length product was 896.7 ± 129.9 mGy × cm.

### Calculation of cortical thickness [23, 24]

Cortical thickness of the tibial diaphysis was automatically calculated in a three-dimensional space using high-resolution measurement reported by Treece et al. [23, 24] (Figs. 1 and 2), which allows for accurate estimates of cortical thickness based on an estimate of cortical density. This software has been primarily used to analyze the cortical thickness of the femoral neck because the hip fracture has consisted in problems in clinical practice [3, 23]. The technique was implemented using Stradwin software (version 5.3; Medical Imaging Group, Machine Intelligence Laboratory, Cambridge Engineering Department, Cambridge, UK), which is available for free download and is a new tool with a demonstrated subvoxel accuracy in assessing cortical bone properties using routine low-resolution CT. The method uses a complex model-based fit approach using the mathematical model of the anatomy and imaging system, calculates from thousands of data points across the bone surface, and assesses using semi-automatic segmentation [23]. The creation of the surface and use of surface normals to guide thickness estimation were performed as follows (Figs. 1 and 2): a surface was generated by thresholding the entire dataset and extracting the contours in each plane to subpixel resolution. Then, contours were edited locally to correct erroneously excluded regions and remove adjoining structures. A surface was interpolated through these contours, and surface vertices and normals were used to guide in-plane thickness estimates by a mathematical equation. The number of measurement points per subject was 5000–9000, depending on the tibial length. Cortical thickness was calculated for each point. Given prior segmentation of the tibia, CT values (Hounsfield units) were examined along the short lines that straddle and are perpendicular to the cortex. Once cortical thickness was estimated at each vertex, this was mapped back onto the surface as a color using a cortical bone mapping technique (Fig. 2). A high-resolution thickness map was filtered over the surface.

Regarding accuracy, Treece et al. [23] tested the validity of the constant-density assumption by measuring the true density of cadaveric femurs in high-resolution CT, which is approximately seven times the resolution of low-resolution scans. They reported that cortical bone thickness estimates were accurate up to 0.3 mm. The technique was also validated in vivo in the context of osteoporosis and hip fractures within laminar structures [3, 23].

### Anatomical tibial coordinate system

**Fig. 4:**
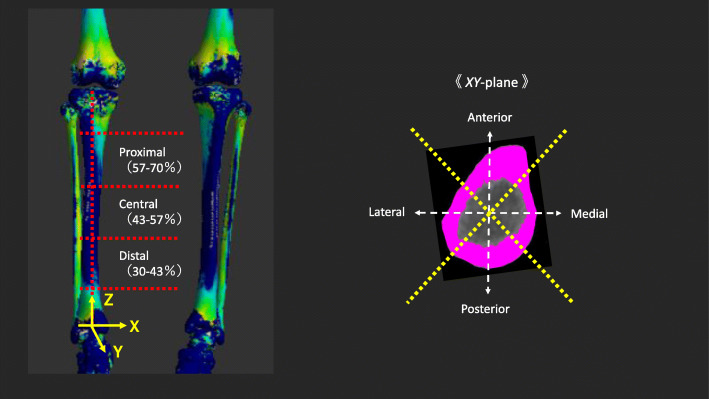
Schematic illustration of the tibial coordinate system and 12 regions divided by each of the three heights (proximal, central, and distal) and four areas (medial, anterior, lateral, and posterior). An anatomical coordinate system of the tibia was constructed. Twelve regions were created by combining three heights (proximal, 57%–70%; central, 43%–57%; distal, 30%–43%) and four areas of the axial plane (*xy*-plane) at 90° (medial, anterior, lateral, and posterior), comprising cortical thickness data from 200 to 1000 points in each region

An anatomical coordinate system for the tibia was constructed using our original software (Fig. 4). First, the three-dimensional CT tibial model was downloaded, and the temporal *z*-axis was defined as the axis connecting two centers of the approximated circles in the tibial diaphysis at two transverse planes. Second, the line connecting the attachment of the posterior cruciate ligament with the medial edge of the tibial tuberosity was defined as the *y*-axis (positive anteriorly). The cross-product of the temporal *z*-axis and *y*-axis was defined as the tibial *x*-axis (positive right). Finally, the cross-product of the *y*-axis and *x*-axis was the true tibial *z*-axis (positive superiorly). The origin point of the tibial coordinate system was defined as the cross-point between the distal tibial articular surface and the true *z*-axis.

### Evaluation parameters

Twelve regions were created by combining three heights (proximal, 57%–70%; central, 43%–57%; distal, 30%–43%) and four areas of the axial plane (*xy*-plane) at 90° (medial, anterior, lateral, and posterior) (Fig. 4). Height was defined as follows: the tibial length was defined from the midpoint of the tibial eminences to the midpoint of the medial and lateral points on top of the talar dome, presenting 100% of the tibial length. The tibial diaphysis was defined as 30%–70%, divided into three heights, and categorized by 17% (proximal, 57%–70%; central, 43%–57%; distal, 30%–43%). Each of the 12 regions (three heights × four areas in the *xy*-plane) comprised cortical thickness data from 200 to 1000 points. The assessment parameter was the average of the cortical thickness data from 200 to 1000 points in each region in the tibial diaphysis, divided by each height and area. The cortical thickness in each of the 12 regions was compared among the four groups categorized by sex and age (young men, young women, elderly men, and elderly women). When data were compared, standardized values, not actual values, were applied. The standardized values divided by the tibial length (cortical thickness/tibial length) were applied because cortical thickness is influenced by body constitution (body weight and height). To standardize the values, as the unit of the values must be identical, the tibial length proportional to the body height, not body weight, was selected.

### Precision and reproducibility through all processes

To ensure precision and test–retest reliability of the measurement of cortical thickness in a three-dimensional space through all processes, two researchers performed two measurements as one set in 10 subjects randomly selected from each group. CT was performed once in each subject so that the precision in this study excluded CT scanning conditions. The precision for cortical thickness of the total diaphysis was calculated as follows:

$$ \frac{\left(\left|1\mathrm{st}-2\mathrm{nd}\right|\right)\mathrm{case}1+\left(\ \right)\mathrm{case}2+\left(\ \right)\mathrm{case}3+\left(\ \right)\mathrm{case}4+\left(\ \right)\mathrm{case}5\cdot \cdot \cdot \cdot \left(\ \right)\mathrm{case}10}{10} $$

Mean differences and 95% confidence intervals (CIs) of the differences in the standardized cortical thickness of the total diaphysis were calculated. The mean and maximum differences were 0.1 × 10^− 3^ and 0.3 × 10^− 3^ for researcher #1 and 0.01 × 10^− 3^ and 0.03 × 10^− 3^ for researcher #2, respectively. The 95% CI of the differences was 0.0 × 10^− 3^–0.1 × 10^− 3^ for researcher #1 and 0.0–0.02 × 10^− 3^ for researcher #2, respectively. In the test–retest reliability (SPSS version 21, SPSS Inc., Chicago, IL, USA), intraobserver reproducibility via the intraclass correlation coefficient of the two measurements was 0.925 (*p* < 0.001) for researcher #1 and 0.998 (*p* < 0.001) for researcher #2. Interobserver reproducibility via the interclass correlation coefficient was 0.989 (*p* = 0.001).

This study was performed based on a protocol approved by the institutional review board of Niigata University. The IRB number was 2015–2351. All subjects provided written or verbal informed consent for study participation and use of their data.

### Statistical analyses

Actual and standardized values are shown in Tables 1 and 2. Comparing the standardized values, one-way analysis of variance (ANOVA) with post hoc test (Tukey) was applied (Tables 3 and 4). In Table 3, the standardized cortical thickness in each height (proximal, central, and distal diaphysis) of the four groups (young men, young women, elderly men, and elderly women) was compared among the four areas of the transverse plane (medial, anterior, lateral, and posterior areas), using one-way ANOVA with Tukey test. In Table 4, the standardized cortical thickness in each of the 12 regions was compared among the four groups categorized by sex and age (young men, young women, elderly men, and elderly women) using one-way ANOVA with Tukey test. Statistical significance was set at a *p*-value < 0.01 (SPSS version 21, SPSS Inc., Chicago, IL, USA). The results of a power analysis for one-way ANOVA among the four groups (young men, young women, elderly men, and elderly women) were as follows: proximal–medial area, power = 1.000, *p* < 0.001; proximal–anterior area, power = 0.266, *p* = 0.392; proximal–lateral area, power = 1.000, *p* < 0.001; proximal–posterior area, power = 0.259, *p* = 0.404; central–medial area, power = 0.574, *p* = 0.077; central–anterior area, power = 0.999, *p* < 0.001; central–lateral area, power = 0.535, *p* = 0.097; central–posterior area, power = 0.999, *p* < 0.001; distal–medial area, power = 0.199, *p* = 0.538; distal–anterior area, power = 0.862, *p* = 0.006; distal–lateral area, power = 0.560, *p* = 0.084; distal–posterior area, power = 0.969, *p* < 0.001.

**Table 1:** Cortical thickness in the tibial diaphysis (actual values)

Actual values (mm)	Young group (*n* = 31)				Elderly group (*n* = 54)			
	Men (*n* = 12)		Women (*n* = 19)		Men (*n* = 29)		Women (*n* = 25)	
	mean	95%CI	mean	95%CI	mean	95%CI	mean	95%CI
Total diaphysis								
Medial	5.7	5.4–6.0	5.1	4.7–5.5	5.7	5.5–5.9	5.6	5.5–5.8
Lateral	6.2	5.5–6.8	4.7	4.5–5.0	5.6	5.4–5.8	5.4	5.2–5.6
Anterior	10.2	8.9–11.5	8.2	7.7–8.8	7.9	7.5–8.2	7.3	7.0–7.6
Posterior	6.4	5.9–6.8	5.8	5.3–6.3	5.3	5.2–5.5	5.0	4.8–5.1
Proximal diaphysis								
Medial	5.1	4.8–5.3	4.6	4.3–4.9	5.6	5.4–5.8	5.5	5.3–5.7
Lateral	5.5	4.9–6.1	4.2	4.0–4.4	5.5	5.3–5.7	5.3	5.0–5.6
Anterior	8.9	7.4–10.3	7.3	6.7–8.0	7.3	7.0–7.7	7.1	6.8–7.4
Posterior	6.2	5.7–6.7	5.4	4.9–5.8	5.3	5.1–5.4	5.1	4.9–5.3
Central diaphysis								
Medial	5.9	5.4–6.4	5.3	4.8–5.8	5.7	5.4–6.0	5.7	5.4–5.9
Lateral	6.6	5.7–7.5	5.1	4.6–5.5	5.4	4.9–5.8	5.4	5.1–5.7
Anterior	11.3	10.0–12.6	9.3	8.6–10.0	8.3	7.8–8.8	7.2	6.5–7.9
Posterior	6.7	6.2–7.2	6.2	5.6–6.7	5.3	5.1–5.5	4.8	4.7–4.9
Distal diaphysis								
Medial	6.3	5.9–6.8	5.9	5.3–6.5	6.1	5.8–6.4	5.9	5.7–6.1
Lateral	6.7	5.9–7.5	5.2	4.8–5.6	5.9	5.5–6.3	5.6	5.3–6.0
Anterior	11.0	9.6–12.3	8.4	7.8–9.0	8.9	8.3–9.5	7.6	7.2–8.1
Posterior	6.2	5.7–6.7	6.0	5.5–6.6	5.6	5.3–5.8	4.8	4.7–4.9

*95%CI* 95% Confidence interval

**Table 2:** Cortical thickness in the tibial diaphysis (standardized values)

Standardized values (×10^−3^)	Young group (*n* = 31)				Elderly group (*n* = 54)			
	Men (*n* = 12)		Women (*n* = 19)		Men (*n* = 29)		Women (*n* = 25)	
	mean	95%CI	mean	95%CI	mean	95%CI	mean	95%CI
Total diaphysis								
Medial	15.7	14.6–16.7	15.9	14.4–17.3	17.3	16.7–18.0	18.1	17.5–18.7
Lateral	17.0	15.0–19.0	14.7	13.8–15.5	17.0	16.3–17.7	17.4	16.6–18.2
Anterior	28.1	24.6–31.7	25.5	23.6–27.4	23.8	22.7–25.0	23.5	22.5–24.5
Posterior	17.5	16.5–18.5	18.0	16.3—19.8	16.1	15.7–16.6	16.0	15.5–16.5
Proximal diaphysis								
Medial	14.0	13.1–14.9	14.1	13.2–15.1	16.9	16.2–17.6	17.9	17.1–18.6
Lateral	15.1	13.3–17.0	12.8	12.3–13.4	16.7	16.1–17.4	17.1	16.2–18.0
Anterior	24.5	20.5–28.5	22.7	20.7–24.7	22.3	21.1–23.4	22.9	21.8–23.9
Posterior	17.1	16.0–18.2	16.7	15.1–18.3	16.0	15.4–16.5	16.4	15.6–17.2
Central diaphysis								
Medial	16.3	14.5–18.1	16.3	14.6–18.0	17.4	16.4–18.4	18.2	17.4–19.1
Lateral	18.3	15.7–20.8	15.6	14.1–17.1	16.2	14.8–17.6	17.4	16.4–18.4
Anterior	31.1	27.6–34.7	28.8	26.4–31.2	25.2	23.7–26.7	23.7	22.5–24.9
Posterior	18.4	17.3–19.6	19.1	17.1–21.2	16.1	15.5–16.6	15.4	15.0–15.9
Distal diaphysis								
Medial	17.5	16.1–18.9	18.3	16.2–20.4	18.6	17.7–19.4	18.9	18.2–19.5
Lateral	18.4	16.2–20.6	16.1	14.9–17.3	17.8	16.7–19.0	18.1	16.9–19.3
Anterior	30.2	26.5–34.0	26.0	24.0–28.0	26.8	25.1–28.6	24.6	23.1–26.1
Posterior	17.1	15.9–18.4	18.7	16.8–20.7	16.8	16.2–17.5	15.6	15.1–16.0

*95%CI* 95% Confidence interval; standardized values means the actual values divided by the tibia length

**Table 3:** Comparison between medial and lateral or between anterior and posterior standardized thickness

		Young men		Young women		Elderly men		Elderly women	
		*p* value	Summary	*p* value	Summary	*p* value	Summary	*p* value	Summary
Total diaphysis	M–L	n.s.	–	n.s.	–	n.s.	–	n.s.	–
	A–P	<.001	A > P	<.001	A > P	<.001	A > P	<.001	A > P
Proximal diaphysis	M–L	n.s.	–	n.s.	–	n.s.	–	n.s.	–
	A–P	<.001	A > P	<.001	A > P	<.001	A > P	<.001	A > P
Central diaphysis	M–L	n.s.	–	n.s.	–	n.s.	–	n.s.	–
	A–P	<.001	A > P	<.001	A > P	<.001	A > P	<.001	A > P
Distal diaphysis	M–L	n.s.	–	n.s.	–	n.s.	–	n.s.	–
	A–P	<.001	A > P	<.001	A > P	<.001	A > P	<.001	A > P

The standardized cortical thickness in each height (proximal, central, and distal diaphysis) of the four groups (young men, young women, elderly men, and elderly women) was compared among the four areas of the axial plane (medial, anterior, lateral, and posterior areas), using one-way ANOVA with Tukey test. M–L = comparison between medial and lateral standardized thickness; A–P = comparison between anterior and posterior standardized thickness; M = medial standardized thickness; L = lateral standardized thickness; A = anterior standardized thickness; P = posterior standardized thickness; n.s. = *p* > .01

**Table 4:** Sex– and age– related differences

		Men (M) vs Women (W) (sex–related difference)				Young (Y) vs Elder group (E) (age–related difference)			
		Young group		Elderly group		Men group		Women group	
		*p* value	Summary	*p* value	Summary	*p* value	Summary	*p* value	Summary
Total diaphysis	M	n.s.	–	n.s.	–	n.s.	–	.002	Y < E
	L	n.s.	–	n.s.	–	n.s.	–	<.001	Y < E
	A	n.s.	–	n.s.	–	.003	Y > E	n.s.	–
	P	n.s.	–	n.s.	–	n.s.	–	.009	Y > E
Proximal diaphysis	M	n.s.	–	n.s.	–	<.001	Y < E	<.001	Y < E
	L	n.s.	–	n.s.	–	n.s.	–	<.001	Y < E
	A	n.s.	–	n.s.	–	n.s.	–	n.s.	–
	P	n.s.	–	n.s.	–	n.s.	–	n.s.	–
Central diaphysis	M	n.s.	–	n.s.	–	n.s.	–	n.s.	–
	L	n.s.	–	n.s.	–	n.s.	–	n.s.	–
	A	n.s.	–	n.s.	–	.001	Y > E	.001	Y > E
	P	n.s.	–	n.s.	–	n.s.	–	<.001	Y > E
Distal diaphysis	M	n.s.	–	n.s.	–	n.s.	–	n.s.	–
	L	n.s.	–	n.s.	–	n.s.	–	n.s.	–
	A	n.s.	–	n.s.	–	n.s.	–	n.s.	–
	P	n.s.	–	n.s.	–	n.s.	–	<.001	Y > E

The standardized cortical thickness in each of the 12 regions was compared among the four groups categorized by sex and age (young men, young women, elderly men, and elderly women), using one-way ANOVA with Tukey test. M = medial thickness; L = lateral thickness; A = anterior thickness; P = posterior thickness; n.s. = *p* > .01

## Results

The actual and standardized cortical thicknesses are presented in Tables 1 and 2. Regarding the structural characteristics in normal individuals (young group) (Table 3), there were no differences in the medial and lateral thicknesses in the proximal, central, and distal diaphysis. The anterior thickness was greater than the posterior thickness in the proximal (men, *p* < 0.001; women, *p* < 0.001), central (men, *p* < 0.001; women, *p* < 0.001), and distal diaphysis (men, *p* < 0.001; women, *p* < 0.001).

In terms of sex-related differences (Table 4), all 12 regions showed no statistically significant differences, whereas elderly women possessed the trend of greater cortical thickness than elderly men in the medial and lateral areas of all heights. Regarding the anterior and posterior areas, elderly women exhibited the trend of the lesser cortical thickness than elderly men in the central and distal heights with no statistically significant differences.

With respect to age-related differences (Table 4), elderly women showed greater cortical thickness than young women in the medial (*p* < 0.001) and lateral areas (*p* < 0.001) of the proximal height, and elderly men showed greater cortical thickness than young men in the medial area of the proximal height (*p* < 0.001). In the anterior and posterior areas, elderly women showed lesser cortical thickness than young women (central–anterior, *p* = 0.001; central–posterior, *p* < 0.001; distal–posterior, *p* < 0.001), and elderly men demonstrated lesser cortical thickness than young men (central–anterior, *p* = 0.001).

## Discussion

The most important finding of the present study was that cortical thickness of the tibial diaphysis reveals age- and sex-related characteristics between non-obese healthy young and elderly subjects depending on the tibial regions. For clinical relevance, the results can be the reference points to clarify the causes of various pathological conditions in lower extremity diseases, such as knee OA.

This study supplied the similar trends observed in young men and women; there were no differences in the medial and lateral thicknesses, while the anterior thickness was greater than the posterior thickness. Cristofolini et al. [25] reported that the geometry of the tibia shows that the diaphysis is shaped so as to resist best to a bending load in a sagittal plane. The linearity was stronger for the area and inertia properties corresponding to a moment in a sagittal plane than in a frontal plane. From the viewpoint of stress fracture, the tibia was the most exposed site for in vivo strain measurements due to accessibility and being a common pathologic site of stress fracture in the lower extremity [26]. The predominant components of force during physiological activities are axial and anteroposterior directions [27]. While axial force generates a state of axial compression, the anteroposterior force component generates a bending moment, which can cause large stress. To adapt itself to large stress, the tibia becomes approximately 50% stiffer in a sagittal plane (bending stiffness, 5.989 Nm/mm) than in a frontal plane (3.588 Nm/mm) [28]. This study’s result indicates that the greater anterior cortical thickness is indispensable to resist the bending moment by the anteroposterior force.

Sex-related differences were not shown at all regions in young and elderly individuals. In the longitudinal study from childhood to early adulthood, sex differences in the distal tibia and radius of growth-related adaptations in bone microarchitecture, geometry, density, and strength across adolescent growth were shown [29]. In the femur, the sex-related differences demonstrated that cortical thickness was greater in men than in women, depending on the region of the whole femur [1]. The study to assess the cortical porosity and thickness of the tibia, using high-resolution CT and linear finite element analysis, exhibited that regional porosity and thickness provided increased sensitivity to sex differences compared to the global analysis at the tibia (− 17% in women) [30]. Sex-related difference in cortical thickness varies in each region of each bone, depending on many factors, especially related to biomechanics, osteoporosis, and hormones [1, 29–31], whereas the tibia possessed similar thickness in all regions for either young or elderly individuals in this study.

Regarding the age-related difference, elderly individuals showed greater cortical thickness in the medial and lateral areas of the proximal diaphysis than young individuals. In the medial–lateral side in the coronal plane, lower leg alignment may be associated with this. In normal subjects, elderly individuals possess slight varus alignment compared to young individuals [32]. Varus coronal alignment is correlated with the inclination of the medial compartment of the proximal tibia [10]. The structure of the proximal tibial metaphysis is mechanically weak due to its low bone mineral density and has laterality between the medial and lateral knee compartments [33, 34]. The laterality of bone mineral density can affect the proximal tibial inclination; thus, the proximal tibia is the key factor for deformation by aging and knee OA [5, 10, 32]. The result of this study, showing greater thickness in the medial and lateral areas of the proximal diaphysis, may explain the remodeling of the proximal tibia due to aging. In terms of remodeling, the periosteum side and medullary cavity side switch to either the compression side or tension side [35]. Remodeling includes “bone formation by compression” and “bone resorption by tension.” Load bearing in the medial proximal tibia works at the medial cortical bone as “compression” in the periosteum side and “tension” in the medullary cavity side. In contrast, at the lateral cortical bone of the proximal tibia, it works as “compression” in the medullary cavity side and “tension” in the periosteum side. Then, it is probable that cortical thickness in not only the medial area but also the lateral areas of the proximal diaphysis is greater in elderly individuals by remodeling. This age-related difference implies that the medial proximal tibia gradually deforms (inclines medially) with aging. Higano et al. [5] demonstrated that the greater medial cortical thickness in the proximal tibia before the development of knee OA was associated with its progression. Their finding indicates that cortical thickness can be the index of load distribution and its related deformity. As our hypothesis, it is assumed that the consecutive mechanical burden in the medial compartment of the proximal tibia throughout life gradually causes deformation, including the change in cortical thickness with aging, such as medial inclination of the proximal tibia and varus alignment, and leads to the development and progression of varus knee OA.

Elderly individuals have lesser thickness of the anterior and posterior areas than young individuals (Tables 1 and 2). Gradual bone loss is common with aging. In women, the prevalence of osteoporosis rapidly increased at the age of 70 years. In men, the prevalence increased at the age of 80 years [31]. The correlation between cortical thickness and bone mineral density, which is the parameter of osteoporosis, has also been reported [36]. Lesser anterior and posterior cortical thicknesses in elderly individuals in the present study may be due to osteoporosis and its related factors, rather thickening by remodeling. As structural characteristics, the tibial diaphysis in the sagittal plane (anteroposterior plane) is originally stronger than that in the coronal plane [25] so that the macrostructural deformity in the sagittal plane, such as bony bowing, may less likely to occur, leading to less remodeling by deformation in the tibia.

The introduction of this evaluation in clinical practice has the disadvantage of a radiation exposure from CT-scan. The potential adverse effects of radiation exposure have become a concern [37, 38]. As radiation exposure was similar to that of common clinical settings, it did not directly cause adverse effects. However, healthy young women were exposed to radiation at a part of their pelvises for this study; hence, they need to be carefully observed. In the future study, one protocol for CT should reduce radiation dose while maintaining image quality.

### Limitations

This study has limitations. First, the accuracy of this method depended on osteoporosis and CT scanning conditions, such as pixel and voxel size. Treece et al. [23] demonstrated a high degree of accuracy using the lesser cortical thickness of the femoral neck [3, 23]. This study’s use of greater cortical thickness of the tibial diaphysis than that of the femoral neck can be assumed to be effective. Additionally, the size ranges of the pixels and voxels in this study were relatively narrow, limiting the effect of the CT scanning conditions on calculations of cortical thickness and reconstruction of 3D models. Second, while the statistical power of this study was sufficient, the sample size was not large, especially in young men. Third, although the regions were strictly divided based on a coordinate system, bony torsional deformities may have influenced the demarcation of the regions and study results. All subjects were healthy so that the rotational effect is likely minimum. Fourth, precision obtained via all processes in the current study excluded CT scans because radiation exposure would increase two or three times if the CT data for each subject were acquired repeatedly, which is not ethically permissible for a research study. Lastly, osteoporosis was not assessed using dual-energy X-ray absorptiometry for elderly individuals, making it impossible to accurately evaluate any association between cortical thickness and osteoporosis. Further assessments should be included in future studies.

With regard to clinical relevance, these normal subject data have the potential to become reference points to clarify the causes of various pathological conditions in lower extremity diseases, such as knee OA.

## Conclusions

Age- and sex-related characteristics in cortical thickness of the tibial diaphysis between non-obese healthy young and elderly subjects are different depending on the tibial regions. The results of this study are of clinical relevance as reference points to clarify the causes of various pathological conditions for diseases.

## Data Availability

References see PubMed.gov.

## Abbreviations

OA: Knee osteoarthritis
CT: Computed tomography
SD: Standard deviation
BMI: Body mass index
CI: Confidence interval
IRB: Institutional review board
ANOVA: One-way analysis of variance
